# Time-decay patterns and irregular disturbance: contrasting roles of abundant and rare microbial communities in dynamic coastal seawater

**DOI:** 10.1128/aem.01751-24

**Published:** 2024-12-09

**Authors:** Yulin Zhang, Derui Song, Peng Yao, Xiao-Hua Zhang, Jiwen Liu

**Affiliations:** 1 Frontiers Science Center for Deep Ocean Multispheres and Earth System, College of Marine Life Sciences, Ocean University of China 12591 , Qingdao, China; 2 School of Computing Sciences, University of East Anglia 151458 , Norwich, United Kingdom; 3 Laboratory for Marine Ecology and Environmental Science, Qingdao Marine Science and Technology Center, Qingdao, China; 4 Key Laboratory of Marine Chemistry Theory and Technology, Ministry of Education, Ocean University of China, Qingdao, China; 5 Key Laboratory of Evolution & Marine Biodiversity (Ministry of Education) and Institute of Evolution & Marine Biodiversity, Ocean University of China 535359 , Qingdao, China; University of Delaware, Lewes, Delaware, USA

**Keywords:** abundant and rare communities, time-decay pattern, assembly process, coastal seawater

## Abstract

Microbial communities in coastal seas experience strong environmental disturbances, yet their response patterns, especially regarding differently abundant subcommunities, remain poorly understood. Here, through 16S rRNA gene amplicon sequencing, we investigated the diversity, time-decay pattern, and assembly process of abundant, conditionally rare taxa (CRT) and rare microbial subcommunities in temperate coastal waters over 60 consecutive weeks. The abundant (50.9%) and CRT (46.1%) communities each comprised approximately half of the planktonic community, while the CRT and rare communities contributed to the extremely high species diversity. Distinct temporal heterogeneity was observed among the three fractions and was associated with taxonomic level. The abundant subcommunity exhibited time-decay patterns at all taxonomic levels, while for CRT, the pattern was found only at finer levels. In contrast, variations of the rare community loosely followed a temporal rhythm and were largely confined within a specific taxonomic range, likely raised from turnovers among closely related taxa. Determinism dominated the community assembly of the abundant fraction, while the rare one was more controlled by stochasticity that may be related to pulse terrigenous inputs and anthropogenic disturbances. The rare subcommunity with narrow niche widths likely represented a stable repository to offer episodic specialists, while the abundant taxa that exhibited broader niche widths were considered the generalists in fluctuating environments. Our study revealed the distinct strategies that abundant and rare communities adopt to maintain community stability in temporal dynamics of prokaryotic plankton in the coastal seawater.

**IMPORTANCE:**

The relative importance of rare and abundant taxa in microbial temporal patterns remains debated. Here, we identified taxonomically associated distinct diversity modes of abundant and rare subcommunities from a year-round time-series study in dynamic coastal seawater. We highlighted the significance of the rare subcommunity in maintaining community stability by serving as a repository to offer specialists driven by stochastic processes over time. The abundant subcommunity, by contrast, contributed mainly to temporal rhythmic variations. This study expands the current understanding of the temporal dynamics and stability of coastal microbial communities by revealing distinct variation patterns of subcommunities with different abundances.

## INTRODUCTION

Coastal seas are the most closely related area in the ocean linked to human activities. Severe environmental stresses including increasing nutrient and organic inputs have resulted in deteriorated water quality and eutrophication in the coastal ecosystems (1–3). As a result, blooms of algae and phytoplankton frequently occur, affecting the balance of the oceanic system (4, 5). Microorganisms, the cornerstone of the marine food web (6) with great abundance and short generation time, can react quickly to physicochemical gradients and environmental disturbance (7–11). This led to strong dynamic fluctuations and discernible spatiotemporal distribution patterns of microbial communities. Investigation of the temporal microbial dynamics in coastal seawater has provided great insights into microbial-mediated biogeochemical cycling processes (12, 13). To further detail the overall distribution patterns, the complex microbial community can be differentiated into several subcommunities based on their characteristics (14). For example, increasing evidence has shown the differential spatial distribution of differently abundant communities, e.g., abundant and rare subcommunities (15–17). The rare taxa encompass various categories, including conditionally rare taxa (CRT), permanently rare taxa (PRT), and transiently rare taxa (TRT) (18, 19). The classification criteria for these taxa are not standardized, and their contributions in terms of the distribution pattern, environmental response, and community stability remain under debate. This highlights the need for more in-depth studies, e.g., clarifying their temporal patterns and potential functional impacts under complex environmental gradients.

Unraveling the factors that drive community structure and succession in response to environmental changes is a central goal in ecology. It is widely accepted that deterministic and stochastic processes jointly influence the assembly of microbial communities (20). Following this theoretical framework, previous studies conducted in marine environments have revealed the spatial/temporal patterns of microbial communities (e.g., distance decay and seasonality) and the underlying mechanisms. However, while the mechanisms influencing community structure have been extensively studied, the detailed temporal patterns (ecological succession) and the processes driving them remain poorly understood. A previous study conducted with a fluidic ecosystem model showed the community responses and resilience to nutrient addition and environmental perturbation and found that the contributions of determinism and stochasticity are dynamic rather than static (21). Natural ecosystems, with their inherent complexity and constant change, are far more intricate than artificial models. Our previous study revealed that in variable coastal environments, microbial communities exhibited a coexistence of deterministic year-round shifts and stochastic fluctuations (22). However, it remains crucial to deconstruct these mixed dynamics to understand the underpinning mechanisms.

As an important issue in microbial ecology, the stability of microbial communities determines their ecosystem functioning. Community stability is closely related to diversity, niche width, and community assembly processes. In the progress of community change with environmental variations, the ecological niches occupied by different taxa could be distinct and dynamic (23–25). Both abundant and rare subcommunities are indispensable in maintaining community stability, but contrasting results are obtained regarding their responses to environmental disturbances. Several studies showed that abundant subcommunities usually have higher ecological niche widths to utilize a wider range of resources, leading to better adaptation to environmental disturbances and higher stability than that of rare subcommunities (26–28). Conversely, other studies argued that rare subcommunities are steadier under environmental disturbances, likely owing to their higher species richness (3, 29, 30). These inconsistencies may have arisen from nonlinear correlations between diversity and community stability (31). Therefore, finer-scale data may be required to characterize the dynamics of microbial communities in changing environments to understand the community stability and underlying reasons.

To explore the succession mechanisms of abundant and rare subcommunities and their contributions to ecosystem stability under changing environments, we collected coastal seawater samples for 60 successive weeks and performed 16S rRNA gene high-throughput sequencing. We aimed to figure out (i) the diversity and community dynamics of the subcommunities in high-precision time-series; (ii) the community stability traits of the subcommunities along time at different taxonomic levels; (iii) mechanisms underpinning the succession of distinct subcommunities in the fluctuating coastal sea. Our study explored the dynamics of rare and abundant subcommunities and their response to environments in time-series and provided further insights into the essential roles that microorganisms play in coastal ecosystems.

## RESULTS

### General environmental characterization

Weekly detection revealed the seasonal trends and inter-week variations of environmental parameters in coastal surface seawater (Table S1). The successive seasonal variations in temperature, conductivity, and DO were clearly observed. Temperature ranged from 3.5 to 27.6°C, with the highest value in August and the lowest in January. Conductivity displayed the same trend with temperature, while DO had the opposite, with values peaking in winter. After this, inorganic nutrients (NO_3_^-^, NO_2_^-^, SiO_3_^2-,^ and PO_4_^3-^) showed an apparent decrease in December and January. Despite the seasonal trends, the majority of the environmental parameters showed week-to-week fluctuations, reflecting the instability of the coastal seawater environment. The detailed environmental data have been reported in our previous publication (22).

### Characteristics of abundant, CRT, and rare subcommunities

A total of 6,838 amplicon sequence variants (ASVs) were obtained from the high-throughput 16S rRNA gene sequencing of the 60-week samples, of which 197 ASVs were classified into the abundant subcommunity, 2,659 to CRT, and 3,982 to the rare subcommunity, according to their relative abundance (Table S2). The relative and absolute (calculated based on cell counts) abundance of ASVs was significantly different among these three subcommunities (Fig. S1a and S2). The abundant and CRT subcommunities collectively constituted 50.9% and 46.1% of the total planktonic community, respectively (Table S2). The majority of ASVs in these two subcommunities exceeded the relative abundance threshold (1% for abundant and 0.1% for CRT) only for a short period (Fig. S1b); however, the relative proportion of these two subcommunities remained stable over time-series. Analysis of the alpha diversity showed that the CRT had the highest species richness, followed by rare and abundant ones (*P* < 0.01, Wilcoxon rank-sum test) (Fig. S3). The lowest value of the abundant subcommunity referred to a simple composition.

**Fig 1:**
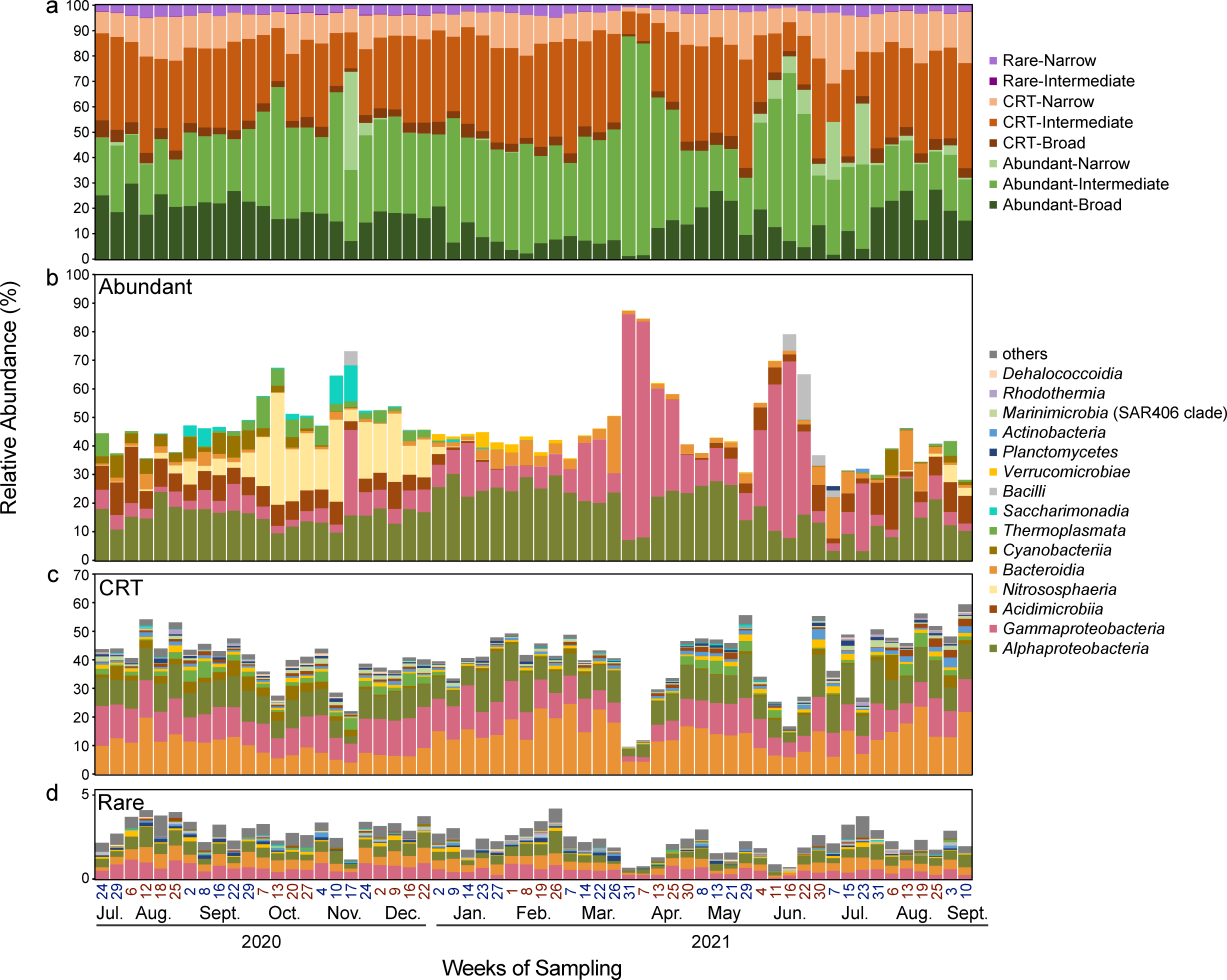
Community compositions of the abundant, CRT, and rare subcommunities. (**a**) The community composition grouped by relative abundance and frequency of occurrence. abundant ASVs: above or equal to 1% relative abundance in at least 1 week; CRTs: ASVs with a relative abundance ≥0.1% in at least 1 week and always < 1%; rare ASVs: always below 0.1% in all samples; broad ASVs: ≥75% occurrence, intermediate ASVs: > 10% and < 75% samples; narrow ASVs: ≤ 10% samples. (**b–d**) The community compositions of abundant, CRT, and rare subcommunities, respectively.

To examine the occurrence frequency, we divided the ASVs into three types, i.e., broad (occurrence in ≥ 75% samples), intermediate (> 10% and < 75%), and narrow (≤ 10%) distributed ASVs (Fig. 1a). Most of the ASVs (5,833/6,838) were narrowly distributed over time, making up 16.9% of the total community. Intermediate ASVs dominated the community with a relative abundance of 64.6%, with abundant and CRT ASVs each contributing equally to this proportion. These results indicated that the microbial community had high diversity, particularly within the CRT and rare subcommunities. Moreover, these subcommunities may change over time due to the presence of impermanent ASVs.

### Community structure and taxonomic distribution of distinct subcommunities

The three subcommunities exhibited distinct taxonomic compositions (Fig. 1b through d; Fig. S4). The abundant subcommunity was represented by *Alphaproteobacteria*, *Gammaproteobacteria*, and *Acidimicrobiia*, while *Bacteroidia*, together with *Alphaproteobacteria* and *Gammaproteobacteria,* dominated the CRT and rare subcommunities. The distribution of some major taxa showed a clear preference toward different subcommunities (Fig. S5). *Bacteroidota* showed a significant preference toward CRT (*P* < 0.001, Welch’s *t*-test). Meanwhile, *Alphaproteobacteria, Crenarchaeota* (Greengenes2: *Thermoproteota*)*,* and *Actinobacteriota* (Greengenes2: *Actinomycetota*) were more prevalent in the abundant subcommunity (*P* < 0.001, Welch’s *t*-test). In contrast to the year-round consistency of the CRT and rare subcommunities, abundant subcommunities showed more seasonal variations at the broad taxonomic level (Fig. 1b through d; Fig. S4). Taxa such as *Nitrososphaeria*, *Cyanobacteria*, and *Thermoplasmata* (Greengenes2: *Poseidoniia*) temporarily occurred in the abundant community during July–December, while *Proteobacteria* (Greengenes2: *Pseudomonadota*) was the main taxon in the rest of the time (Fig. 1b). At the genus level, genera shared across all three subcommunities accounted for 21.2%–83.7% of the total community, with the proportion being lower between January and March (Fig. S6). The predominant overlapping genera included the SAR11 clade (Greengenes2: *Pelagibacter*), *Actinomarina*, and *Ralstonia*, as well as two archaeal taxa (*Nitrosopumilus* and Marine Group II).

### Distinct temporal variation patterns of subcommunities

**Fig 2:**
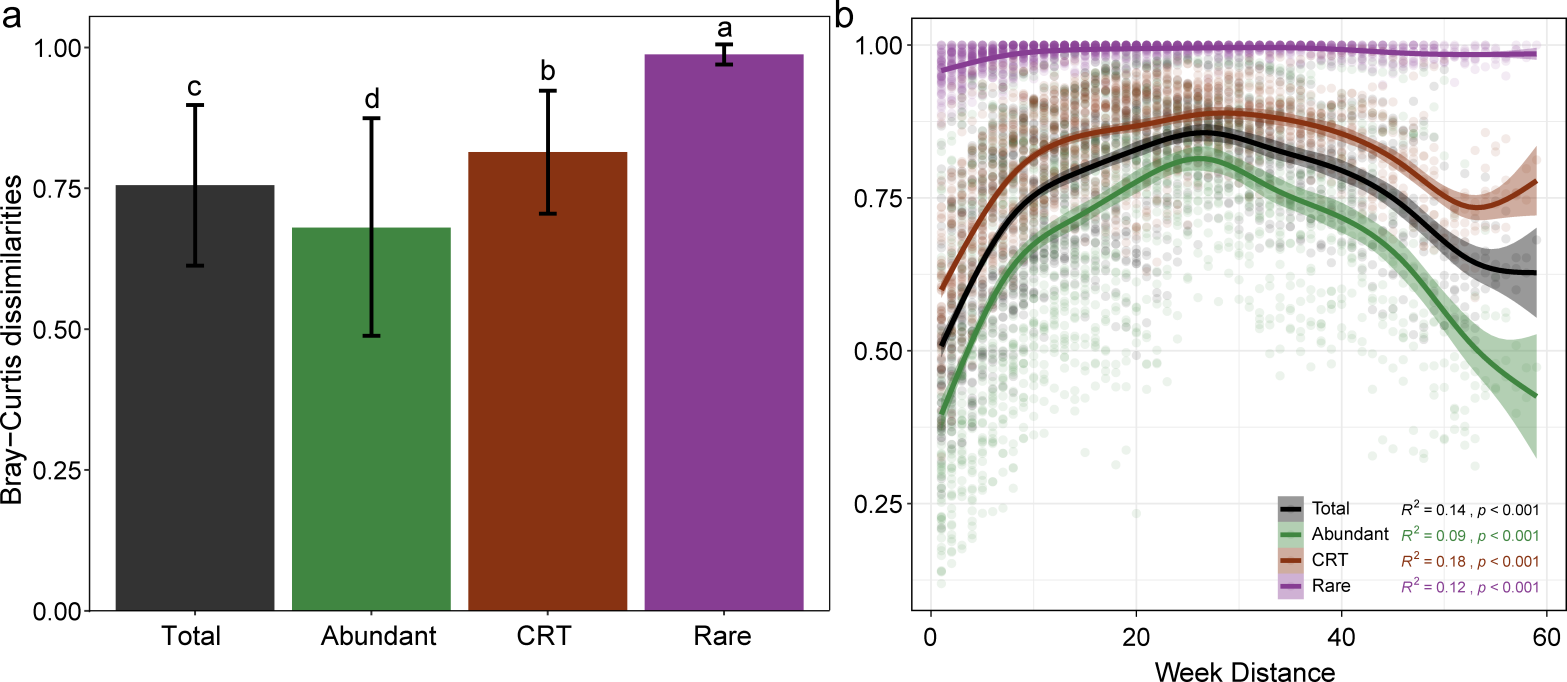
The Bray–Curtis dissimilarities of different subcommunities. (**a**) Wilcoxon rank-sum tests were carried out to examine the significance of differences between groups; (**b**) Correlations between Bray–Curtis dissimilarities and time distances at ASV level in the three subcommunities and total community. The correlation was fitted with a generalized additive model (GAM), with the shaded areas representing 95% confidence intervals. Spearman’s correlation coefficient (R) and *P* value were used to evaluate the significance of the correlation between week distance and Bray–Curtis dissimilarities.

**Fig 3:**
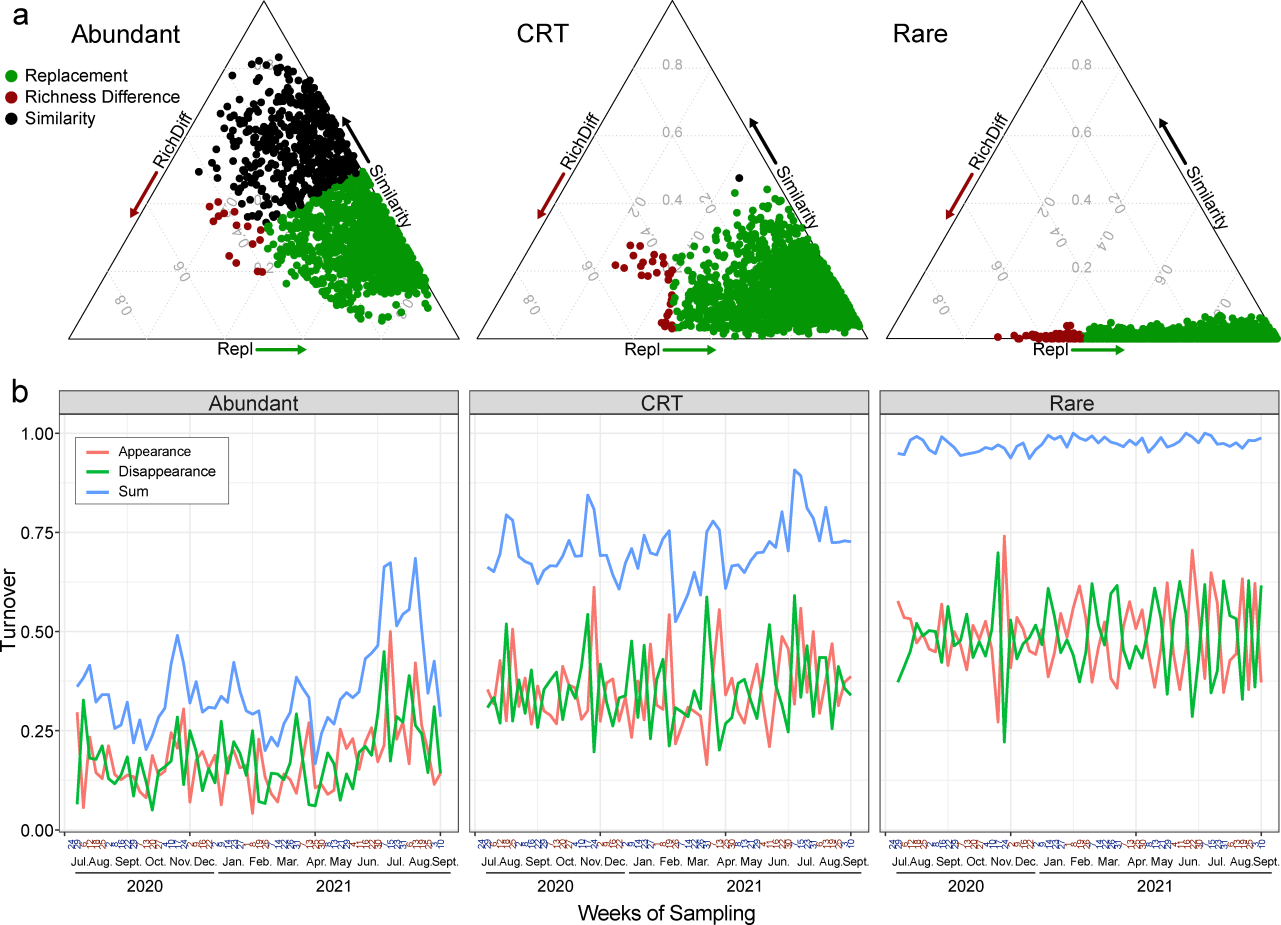
The beta diversity decomposition and turnover analysis of abundant, CRT, and rare subcommunities and total communities. (**a**) the beta diversity decomposition; (**b**) turnover indices. The red, green, and blue lines represent ASV appearance, disappearance, and the summary, respectively.

To further investigate community variations over time, we compared the temporal dynamics of the three subcommunities using Bray–Curtis and unweighted UniFrac distances. The greatest temporal dissimilarities were observed within the rare subcommunities, whereas the lowest dissimilarities were evident in abundant ones (Fig. 2a; Fig. S7a). More importantly, the rare subcommunity exhibited contrasting temporal variation patterns from the other two. While between-sample dissimilarities consistently remained high in the rare subcommunity, they were significantly correlated with temporal (week) distance for the abundant and CRT communities, showing a U-shape variation peaking at a time interval of ~25 weeks (Fig. 2b; Fig. S7b). As revealed by non-metric multidimensional scaling (NMDS) analysis, the highly dispersed distribution corroborated the dissimilarities of the rare subcommunity across all samples, implying temporal disorderliness in this fraction (Fig. S8). With the decomposition of beta diversity and the turnover indices, we found species replacement, rather than richness difference, as the primary component contributing to community variations (Fig. 3).

**Fig 4:**
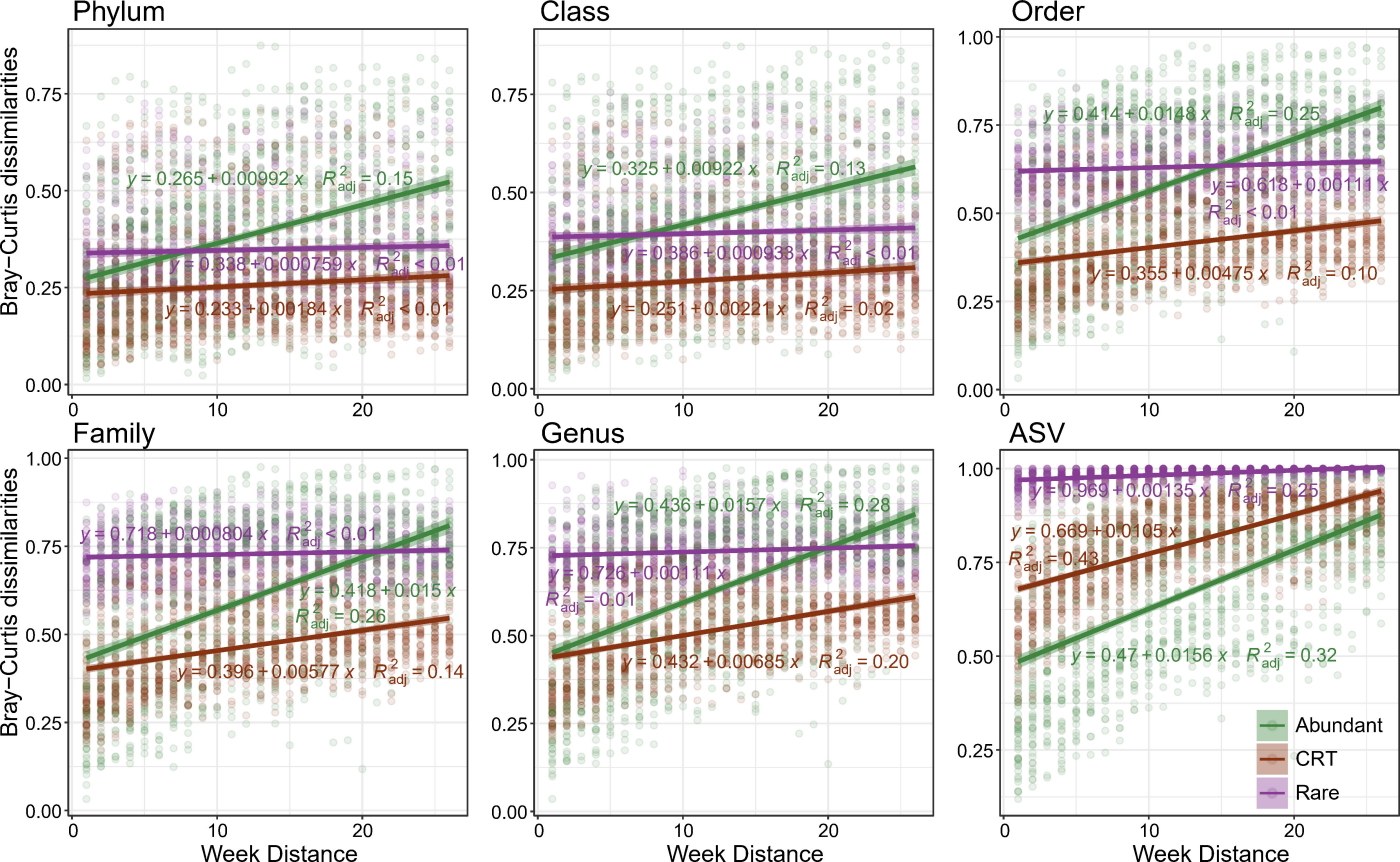
Correlations between Bray–Curtis dissimilarities and time distances at multiple taxonomy levels in the three subcommunities. Spearman’s rank correlations were calculated, with the shaded areas representing 95% confidence intervals.

As observed above, both abundant and rare subcommunities showed consistent temporal patterns at broad and ASV taxonomic levels. However, the CRT subcommunity exhibited varying temporal patterns across different taxonomic levels, being stable at the broad taxonomic level while orderly changing at the ASV level. To understand the origins of these disparities, we examined the temporal pattern at different sequence similarities. The abundant subcommunity showed significant time-decay patterns across all taxonomic levels, whereas the rare one did not show such patterns (Fig. 4). However, the time-decay pattern of CRT diminished progressively with broader taxonomic levels, as indicated by lower slope values. Temporal specificity analysis further supported these findings by showing pronounced temporal specificity at finer taxonomy for CRT (Fig. S9). Considering community dissimilarities (Fig. S10), temporal decay patterns, or temporal specificity, the most drastic transitions occur between the genus and ASV levels. These results suggest that fluctuations in closely related CRTs affected the temporal distribution patterns of this community.

### Ecological processes underlying disparities of temporal patterns

**Fig 5:**
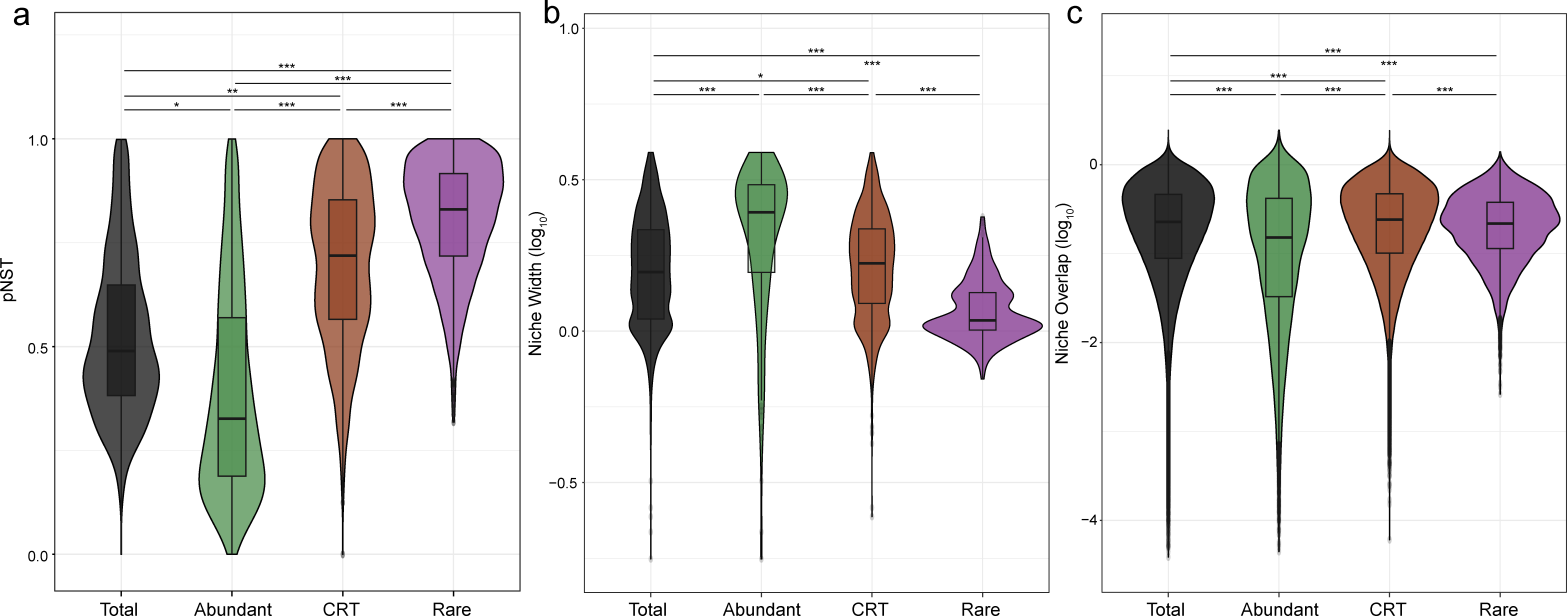
The differences in the community features of the subcommunities and the total community. (**a**) pNST value; (**b**) niche width (log_10_-transformed); (**c**) niche overlap (log_10_-transformed). The Wilcoxon test between groups was marked by stars (***: *P* < 0.001, **: *P* < 0.01, *: *P* < 0.05, ns: not significant).

To explore mechanisms underpinning the distinct temporal patterns of the three subcommunities, we evaluated the relative roles of deterministic and stochastic processes using the phylogenetic normalized stochasticity ratio (pNST). The three portions were driven by different ecological processes (*P* < 0.001, Wilcoxon rank-sum test) (Fig. 5a). The lowest pNST value of the abundant portion indicated a strong influence of deterministic processes, whereas the rare subcommunity was primarily governed by stochasticity according to the relative higher pNST value. The CRT was primarily influenced by stochastic processes, although determinism also had a significant contribution.

**Fig 6:**
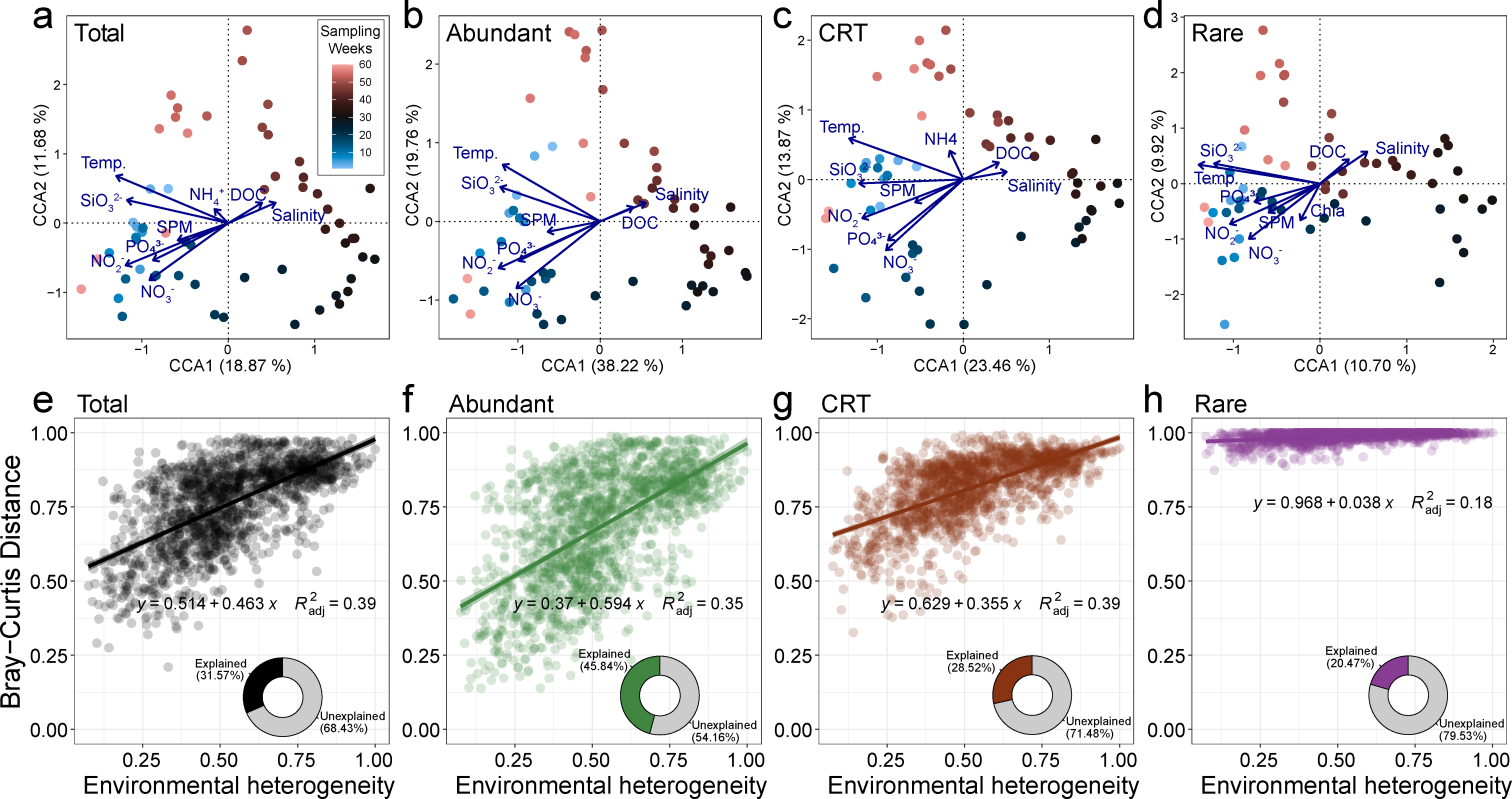
The relationship between environmental factors and total, abundant, CRT, and rare subcommunities. (**a–d**) CCA for the communities. (**e–h**) Fitting between Bray–Curtis dissimilarity and environmental heterogeneity. The environmental heterogeneity was calculated with a Euclidean distance matrix, based on all 11 measured variables.

CCA analyses showed that the explanatory power of environmental factors was highest for the abundant subcommunity (45.8%), moderate for the CRT (28.5%), and lowest for the rare portion (20.5%). The temporal dynamics of the three subcommunities were affected by similar factors, with temperature, silicate, NO_3_^-^, and PO_4_^3-^ showing the major effects (Fig. 6a through d). Compared to other portions, the rare subcommunity had greater correlations with chlorophyll a (*P* < 0.001) and salinity (*P* < 0.05) and a weaker correlation with temperature according to Mantel’s test. CRT and rare subcommunities were more influenced by NO_2_^-^ and PO_4_^3-^ compared with the abundant portion (Table S3). By calculating the environmental heterogeneity of the 11 measured environmental factors and analyzing their correlations with the Bray–Curtis dissimilarities, it was revealed that the abundant subcommunity appeared to be more sensitive to environmental changes. CRT was affected to a lesser extent, whereas the temporal dynamics of the rare portion exhibited a minimal association with the environmental variability (Fig. 6e through h).

To assess the contribution of ASVs of the three subcommunities in maintaining the temporal stability, we conducted ecological niche width and overlap analyses (Fig. 5b and c). As a result, the niche width of abundant, CRT, and rare ASVs decreased gradually, indicating the best adaptation of abundant ASVs to the environments. However, the high ecological niche overlap of the CRTs suggested that multiple CRTs may coexist under the same environmental conditions.

## DISCUSSION

In this study, we explored the temporal dynamics of taxa with different relative abundances in planktonic microbial communities by collecting high-resolution time-series samples from coastal seawater. We found distinct temporal distribution patterns among abundant, CRT, and rare subcommunities at different taxonomic levels. While the abundant portion contributed to community seasonality, the CRT and rare portions made substantial contributions to the extremely high species diversity and variability at finer taxonomic levels. Assembly of the abundant subcommunity was mainly controlled by determinism, while stochastic processes played a more important role in governing the CRT and rare portions. The three subcommunities, being distinct in diversity, temporal patterns, and driving forces, collectively maintain the dynamic stability of the community.

Microbial communities typically exhibit a skewed abundance distribution, predominated by a few highly abundant species (18, 32). Our findings support this notion in a temporal dimension, revealing only 197 abundant ASVs constituting 50.89% of the total community. These taxa had broad ecological niche widths, high occurrence frequencies, and robust environmental adaptabilities. In the spatial dimension, it has been reported that generalists rather than specialists may more dominantly affect the beta diversity variations along the trophic gradient (33). However, a study conducted on the temporal dimension emphasized the contributions of the rare biosphere to the seasonality of coastal marine bacterioplankton (34). We confirmed the substantial contribution of rare taxa to temporal variations. However, we observed that their extremely high dissimilarities contributed little to rhythmic community succession throughout the year. Indeed, the rare community exhibited a disordered temporal pattern. We found extremely high dissimilarities and the lowest occurrence frequencies of rare ASVs over time, similar to the spatial abundance distribution pattern of rare taxa across the global surface ocean (35). Unexpectedly, the rare portion emerged as the most stable subcommunity over time at high taxonomic levels, suggesting that its high diversity is maintained by a limited degree of genetic divergence. It is likely that certain deterministic factors select for specific high-rank microbial groups within the rare subcommunity, while the temporal patterns of members within these groups are influenced by stochastic processes. In contrast, rhythmic temporal variations were more pronounced in the abundant subcommunity, where variations occurred at broad taxonomic levels, primarily due to environmental heterogeneities.

To comprehensively capture the microbial temporal patterns, we separated CRT, a specific rare subset that occasionally exhibited higher abundance throughout the time-series, for focused analysis. The analysis of CRT offers additional insights into temporal dynamics. By separating the nonabundant biosphere into the CRT and rare parts, we found time-decay patterns in the CRT, but none in the rare part. Many studies have highlighted the role of CRT taxa in dynamic environments, noting that these taxa often experience phase-specific increases in abundance and thus play crucial roles in community dynamics. For example, Pascaline et al. described the seasonal dynamics of CRT in subtropical reservoirs (36), while Ashley et al. emphasized the importance of the CRT subcommunity in driving temporal changes across different environments (19). Our results confirmed these features and further explored the detailed origins and driving factors of these variations. We found that the temporal specificity of CRT was evident only at lower taxonomic levels, while broad levels exhibited temporally stable compositions. This indicated that the time-decay pattern observed within CRT largely stemmed from replacements among closely related taxa. Additionally, the CRT portion, along with the abundant subcommunity, exhibited a higher occurrence frequency in the coastal water compared to the rare subcommunity, demonstrating strong correlations and dominating the microbial co-occurrence networks (Fig. S11). This suggested that they may play a crucial ecological role in microbial interactions and community stability.

On the other hand, the exceptionally high species diversity of two nonabundant portions should not be neglected. We observed that while the rare subcommunity accounted for the lowest proportion (3.05%), it was 20 times more diverse (3,982 ASVs) compared to the abundant counterpart. Furthermore, the CRT not only exhibited high diversity (2,659 ASVs) but also constituted nearly half of the entire community. This proportion is similar to the findings in previous studies conducted in marine environments, confirming the substantial contribution of nonabundant taxa to the high diversity of microbial communities (37–39). According to the insurance hypothesis proposing that biodiversity can ensure ecosystems against decline in their functioning (40, 41), the diversity of microbial communities is strongly correlated with community stability. The significantly high alpha diversity as well as high beta diversity in time-series rare subcommunities could bring community complementary biological traits and functional redundancy that jointly buffered against environmental disturbances, which also contributes to weak sensitivity to environmental heterogeneity over long time scales (3, 41, 42). In eutrophic coastal seawater, the complicated impacts of human activities could perturb the microbial community (43, 44). Together with our results, we indicate that the nonabundant communities offered numerous possibilities and a stable foundation to the entire planktonic community in the face of the fluctuations over time.

As for the assembly processes that drive the distinct temporal variations, we found deterministic processes predominantly controlled the regular variations of the abundant subcommunity, whereas the rare one was mainly influenced by stochastic processes, undergoing a high degree of random species replacements. The CRT portion was mainly controlled by stochasticity, with determinism also playing an indispensable part, exhibiting environmental correlations and temporal specificity at lower taxonomic levels. Zhou et al. found the great impact of stochasticity and environmental perturbations on microbial community successions in a fluidic ecosystem (21). Our results confirmed these points and further demonstrated that in highly dynamic natural environments, stochastic processes mainly impact the temporal pattern of low-abundance portions. It is believed that high dispersal rates have a homogenizing effect (homogeneous dispersal), leading to low community turnover, whereas low dispersal rates (dispersal limitation), coupled with drift or weak selection, increase community turnover (45, 46). This may be the main reason for the great dissimilarities in the rare subcommunity over time. Due to its low biomass, the rare subcommunity is more susceptible to ecological drift, and/or dispersal limitation, leading to distinct variations in random ecological sampling. These processes may lead to the turnover among closely related taxa or their occurrence at specific times, followed by subsequent environmental selection and enrichment. As for the temporal stability at high taxonomic levels of CRT and rare portions, we infer that it may be raised from the selection of determinism on a larger spatial scale.

One limitation in studying rare subcommunities is the inconsistent definitions used across studies conducted under different environmental conditions or temporal–spatial scales (47). Currently, various definitions exist for rare microbial communities, including (i) permanently rare taxa that maintain extremely low abundance in all samples (48); (ii) transiently rare taxa that occur only in a few samples with low abundance (49); (iii) conditionally rare taxa that shift between rare and abundant in some samples (19). In our study, with the pronounced temporal variability exhibited in the community, no permanently abundant or rare taxa were found. It is important to highlight that we observed significant differences between the transiently rare taxa and CRT in temporal distribution patterns and community assembly. Therefore, attention should be given to distinguishing these groups when focusing on the microbial communities across broad spatiotemporal scales or varying environmental conditions to prevent ambiguity arising from classification.

### Conclusion

Through high-precision time-series analyses, we revealed distinctions in compositional shifts and temporal patterns of microbial communities with different relative abundance in coastal seawater. The abundant subcommunity showed time-decay patterns and had a major contribution to the rhythmic community changes over the year. These abundant taxa were broadly temporal distributed, could react to environmental variations, and were mainly controlled by determinism, whereas the CRT and rare portions were the main contributors to the high species diversity of the entire planktonic community. However, unlike the temporal stochasticity observed in the rare portion, the time-decay pattern in CRT was evident only at finer taxonomic levels. At broader taxonomic levels, both exhibited stable community compositions. The disordered and remarkably high variations shown in the rare portion were limited in a certain taxonomic range. Overall, our findings emphasize the distinct strategies employed by the high- and low-abundance subcommunities to maintain stability in a dynamic environment: the former responds and adapts to environmental changes, while the latter contributes substantially to diversity and serves as a repository of episodic specialists, thereby enhancing the community’s resilience to fluctuations.

## MATERIALS AND METHODS

### Sampling site and sample collection

Seawater samples were collected by speedboat from the site at Qingdao No. 2 Bathing Beach (BB), which was about 500 m offshore (Fig. S12). From 24 July 2020 to 10 September 2021 (a total of 60 weeks), surface water (~20 cm depth) was collected every week by acid-washed polypropylene Nalgene bottles (22). Seawater was pre-filtered by the 3-µm pore size polycarbonate membrane (Millipore Corporation, 47 mm diameter) and then filtered through the 0.22-µm pore size polycarbonate membrane to collect planktonic cells. In total, 60 DNA samples were collected. All the filters were flash-frozen in liquid nitrogen before preservation at −80°C.

In order to record *in situ* environmental factors such as water temperature, salinity, conductivity, dissolved oxygen (DO), and pH, a Hach HQ40d portable multiparameter detector was used. We measured dissolved inorganic nutrients (NO_3_^−^, NO_2_^−^, NH_4_^+^, PO_4_^3−,^ and SiO_3_^2-^) using a continuous flow analyzer (Auto-Analyzer 3, Seal Analytical Ltd., UK) after filtering seawater with 0.45-µm cellulose acetate membranes (50–53). Chlorophyll *a* was measured on cellulose acetate filters extracted with acetone and processed by the spectrophotometric method (54). Samples for dissolved organic carbon (DOC) were filtered through precombusted (450°C, 5 hours) 0.7-µm GF/F glass fiber filters and stored in precombusted tubes at −20°C until analysis. The high-temperature catalytic oxidation (HTCO) method (55) was used with the Shimadzu TOC-L analyzer equipped with an ASI-V autosampler.

For bacterial cell counting, triplicate water samples (4 mL each) were collected and fixed with glutaraldehyde (final concentration: 0.5%). After a 20-minute fixation in the dark, the samples were transferred to liquid nitrogen and stored at −80°C. Samples were diluted to 100 x in Milli-Q water and were stained with SYBR Green I (Molecular Probes, final concentration: 0.01%) in the dark. Cell counting was conducted using a CytoFLEX flow cytometer (Beckman Coulter, USA) (56).

### DNA extraction and sequencing

Genomic DNA was extracted with the DNeasy PowerSoil Kit (QIAGEN, Germany) following the manufacturer’s instructions. We used the Fast Prep-24 Homogenization System (MP Biomedicals, Irvine, CA) to accelerate cell lysis at a speed of 6.0 m s^−1^ thrice (5). The NanoDrop-2000 spectro-photometer was used to assess DNA concentration and quality. The hypervariable V4 regions of the bacterial and archaeal 16S rRNA gene primer 515FmodF (5′-GTGYCAGCMGCCGCGGTAA-3′) and 806RmodR (5′-GGACTACNVGGGTWTCTAAT-3′) were used to amplify genes (57). PCR was performed in triplicates using TransGen AP221-02: *Trans* Start Fast pfu DNA Polymerase (TransGen Biotech, Beijing, China) in a 20-µL mixture containing 4 µL of 5 × FastPfu Buffer, 2 µL of 2.5 mM dNTPs, 0.8 µM of each primer, 0.4 µL of FastPfu polymerase, 0.2 µL of bovine serum albumin (BSA), and 10 ng of template DNA. The following thermal conditions were used: initial denaturation at 95°C for 3 minutes, 29 cycles of 95°C for 30 seconds, 55°C for 30 seconds, and 72°C for 45 seconds, followed by a single extension at 72°C for 10 minutes. Following the manufacturer’s instructions, the amplification products were purified using the AxyPrep DNA Gel Extraction Kit from Axygen Biosciences in Union City, California, and quantified using QuantiFluor-ST from Promega in the United States. Triplicate amplicons were pooled for each sample. Different samples were pooled in equimolar concentrations and sequenced (2 × 300 bp) on an Illumina MiSeq PE300 platform at Majorbio Bio-Pharm Technology (Shanghai, China).

### Data processing and amplicon sequence variant analysis

We obtained 57,031 (31,641–108,791) valid reads per sample, with a total base of 14.4 Gb (8.0–27.5 Gb) and Q30 of each sample > 97.3%. The rarefaction curve was constructed to evaluate richness saturation (Fig. S13). We used the QIIME2 pipeline for processing the raw sequencing data (58). After trimming the barcodes and primers and removing sequences with low quality and short length, the high-quality reads were assigned into ASVs using DADA2 (59). ASVs were taxonomically classified using qiime2 classify-sklearn plugin against the SILVA database (v138, http://www.arb-silva.de) with a 70% identity cutoff. Additionally, the Greengenes2 (http://ftp.microbio.me/greengenes_release/current/) was used to provide the GTDB taxonomies (harmonized with the Living Tree Project) (60). To avoid sequencing errors, absolute singletons were removed for downstream analyses. The chloroplast and mitochondria were also removed. Finally, we obtained 6,838 ASVs across all samples. The sequence counts were normalized to relative abundances within each sample to adjust for differences in sequencing depth among samples.

### Definition of abundant, CRT, and rare taxa

In studies of rare and abundant communities, classification typically involves setting relative abundance thresholds (e.g., 1%, 0.1%, or 0.001%) (61, 62). Given the variability in microbial community structures across different environments, these thresholds should be tailored to the specific characteristics of each community. For our study, the rank-abundance curve was conducted to confirm that the classification was suitable to the distribution tails of our data set. To better explore the different dynamic patterns of microbial communities in highly variable environments, we differentiated conditionally rare taxa from other rare taxa, with the remaining taxa collectively referred to as “rare subcommunity.” Finally, the community was artificially classified into three categories according to the 1% and 0.1% thresholds: (i) abundant ASVs: ASVs with a relative abundance ≥1% in at least 1 week (63, 64); (ii) rare ASVs: ASVs always below 0.1% cutoff; (iii) conditionally rare taxa (CRT): ASVs with a relative abundance ≥0.1% in at least 1 week and always < 1%. As for occurrence, we defined broad (≥ 75% samples), intermediate (> 10% and < 75% samples), and narrow (≤ 10% samples) ASVs based on their frequency of occurrence over 60 weeks (65). Detailed information of ASVs is shown in Table S4.

### Statistical analyses

The Shannon, Simpson, and Pielou alpha diversity indices were determined with the “vegan” package in R (66). The Wilcoxon rank-sum test was performed using the “wilcox.test” function to examine the differences between total, abundant, CRT, and rare communities. For beta diversity, Bray–Curtis metrics were used and NMDS was performed. The correlation between Bray–Curtis dissimilarity and week distance was fitted with the linear model (LM) and generalized additive model (GAM) (67). In addition, unweighted UniFrac distances were also calculated to validate Bray–Curtis analyses with the “phyloseq” package in R (68). To explore the time-specificity of communities, the “specificity” package was used (69). The turnover indices were calculated to assess the shift of ASV between adjacent weeks with the “codyn” package (70). Besides, the community dissimilarities over time were partitioned into species replacement and richness difference with the “adespatial” package.

Canonical correspondence analysis (CCA) was executed to analyze the effects of environmental factors with the Log-Ratio transformed compositional data. Environmental heterogeneity was calculated as previously described (46). A Spearman’s rank correlation between Bray–Curtis dissimilarities and environmental heterogeneities was calculated with the “ggpmisc” package (71). Linear discriminant analysis (LDA) effect size (LEfSe) was performed using the “microeco” package to identify biomarkers in the three subcommunities (72, 73). All these analyses were conducted in R v3.6.2 (74). STAMP (v2.1.3) was also used to confirm the discriminant taxa among subcommunities (75). The differences of major taxa were established between the three subcommunities using Welch’s *t*-test. Network analysis was conducted using the “igraph,” “psych,” and “Hmisc” packages in R. Genera occurring in more than 10% of the samples were used to construct a co-occurrence network (65, 76). To filter valid interactions between genus pairs, Spearman’s correlation coefficients (ρ) > |0.7| and *P* values < 0.01 were set as thresholds. Network visualization was conducted using Gephi 0.9.2 (77).

### Community assembly process and niche width analysis

The contribution of deterministic and stochastic processes to community assembly was quantitatively determined using the phylogenetic normalized stochasticity ratio (pNST) with the R package “NST” (78). The pNST cutoff between deterministic assembly (< 50%) and stochastic assembly (> 50%) was set according to the package introduction. The community data were randomly shuffled 1,000 times to generate a set of null expected communities. In order to infer the environment adaptability and resource utilization of the microbial communities, Levins’ niche width index (79) of all ASVs was calculated, representing the niche width of the community (26, 80). Niche overlap of each ASV pair was also calculated to evaluate the sharing of taxa to the environment. The analysis was conducted using the “spaa” package (81).

## Data Availability

The raw sequence reads that support the findings of this study have been deposited in the NODE (http://www.biosino.org/node) under project number OEP003832 (22). Other data generated or analyzed during this study are included in this published article and its supplemental files.

## SUPPLEMENTAL MATERIAL

Supplemental material aem.01751-24-s0001.pdf Figures S1 to S13; Tables S2 and S3.

Table S1 aem.01751-24-s0002.xlsx Statistical data of environmental factors.

Table S4 aem.01751-24-s0003.xlsx Information for the 6,838 ASVs.

ASM does not own the copyrights to Supplemental Material that may be linked to, or accessed through, an article. The authors have granted ASM a non-exclusive, world-wide license to publish the Supplemental Material files. Please contact the corresponding author directly for reuse.
