# Supplementary material for: Time-decay patterns and irregular disturbance: contrasting roles of abundant and rare microbial communities in dynamic coastal seawater
Source: Appl Environ Microbiol. 2024 Dec 9;91(1):e01751-24. doi: 10.1128/aem.01751-24 (PMC11784082; doi:10.1128/aem.01751-24)
Supplement: Supplemental material — Figures S1 to S13; Tables S2 and S3. [file aem.01751-24-s0001.pdf]

Supplementary information

Time-decay Patterns and Irregular Disturbance: Contrasting Roles of Abundant and Rare Microbial Communities in Dynamic Coastal Seawater

Yulin Zhang[1], Derui Song[1,5], Peng Yao[2,4], Xiao-Hua Zhang[1,2,3], Jiwen Liu[1,2,3*]

[1]Frontiers Science Center for Deep Ocean Multispheres and Earth System, and College of Marine Life Sciences, Ocean University of China, Qingdao 266003, China

[2]Laboratory for Marine Ecology and Environmental Science, Qingdao Marine Science and Technology Center, Qingdao 266237, China

[3]Key Laboratory of Evolution & Marine Biodiversity (Ministry of Education) and Institute of Evolution & Marine Biodiversity, Ocean University of China, Qingdao 266003, China

[4]Key Laboratory of Marine Chemistry Theory and Technology, Ministry of Education, Ocean University of China, Qingdao 266100, China

[5]School of Computing Sciences, University of East Anglia, Norwich Research Park, Norwich NR47TJ, UK

*Author for correspondence:

Jiwen Liu, College of Marine Life Sciences, Ocean University of China, 5 Yushan Road, Qingdao 266003, PR China, Tel/Fax: +86-532-82032721, Email: liujiwen@ouc.edu.cn

Running title: Coastal abundant and rare subcommunities.

This file includes:

Figures S1 to S13

Tables S1 to S4

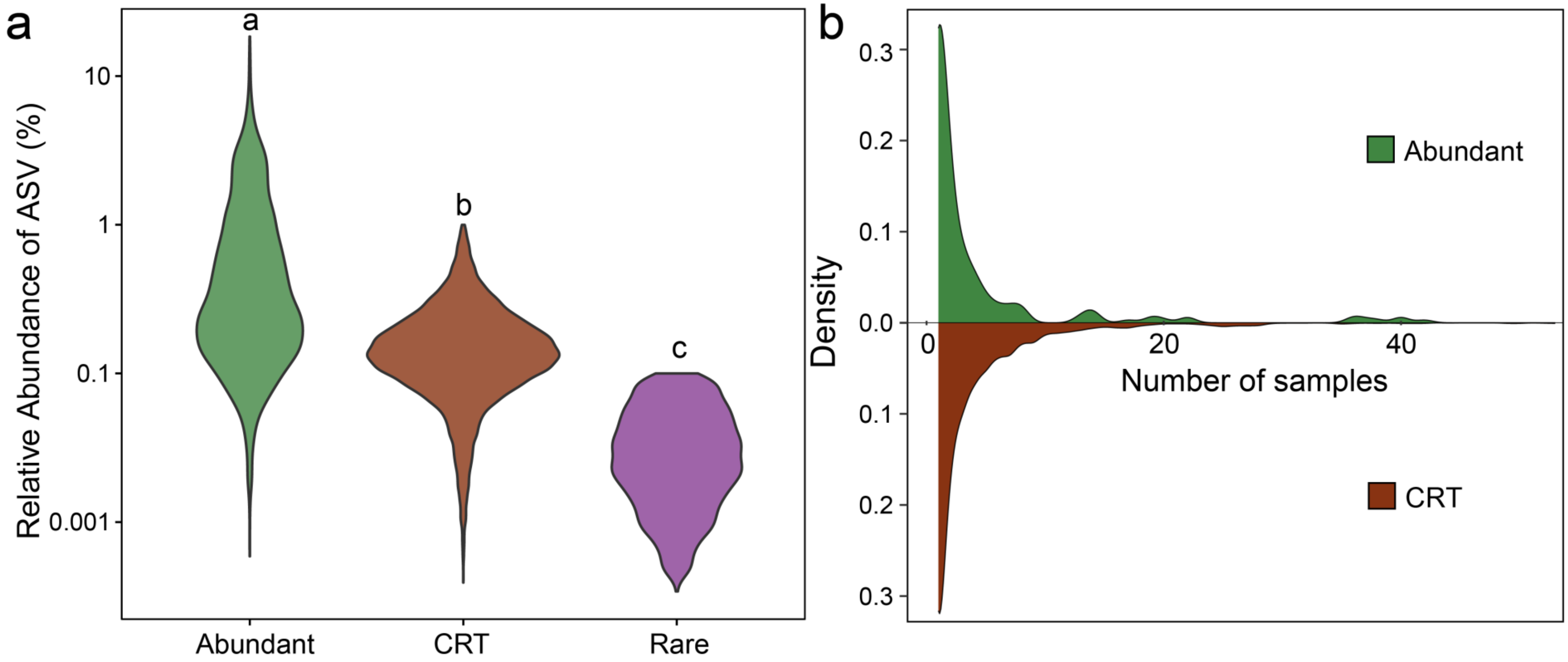

Fig. S1. Relative abundance of ASVs in the three subcommunities and the density plot for abundant and CRT ASVs. (a) Relative abundance of ASVs in the three subcommunities, (b) Density plot showing the frequency (number of samples) with which ASVs exceed the 1% and 0.1% thresholds for the abundant and CRT subcommunities, respectively.

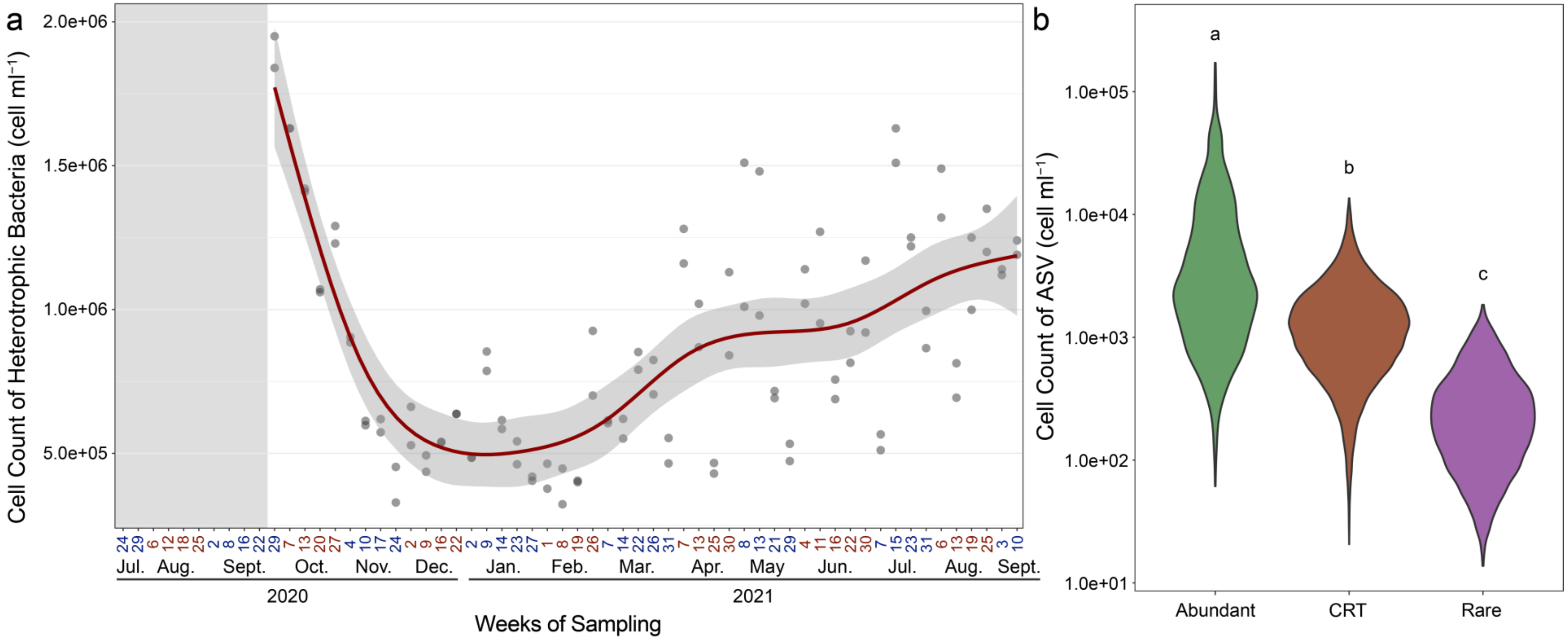

Fig. S2. Cell count of the heterotrophic bacteria (a) and ASVs (b).

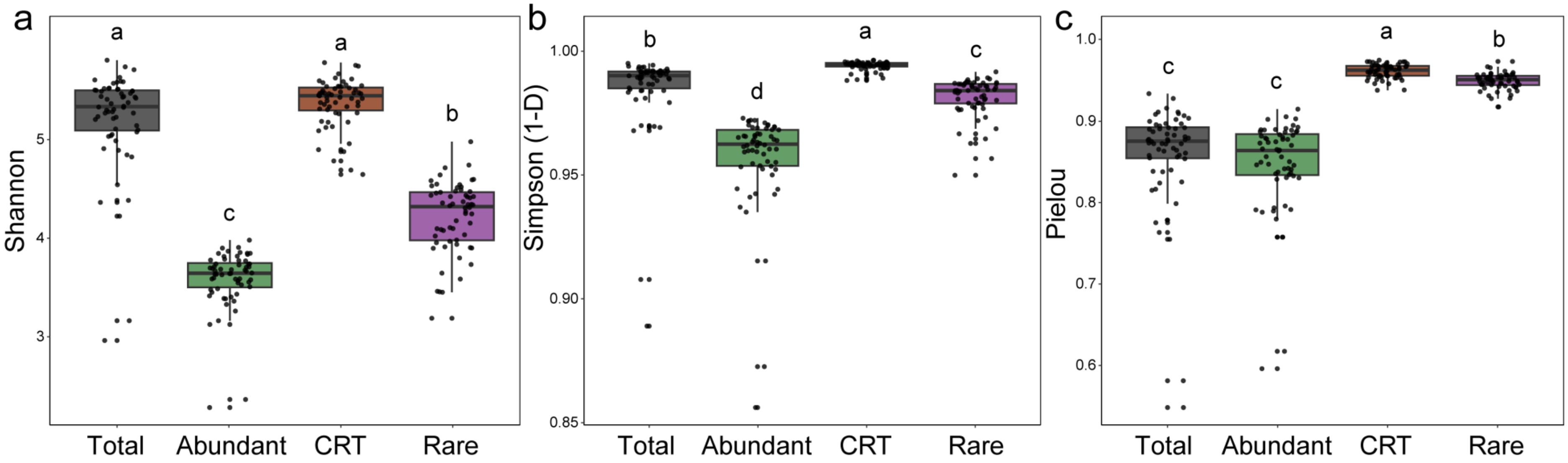

Fig. S3. Alpha diversity indices of the three subcommunities and the total microbial community. (a) Shannon index; (b) Simpson (1-D) index; (c) Pielou index. Wilcoxon rank-sum tests were carried out to examine the significance of differences between groups.

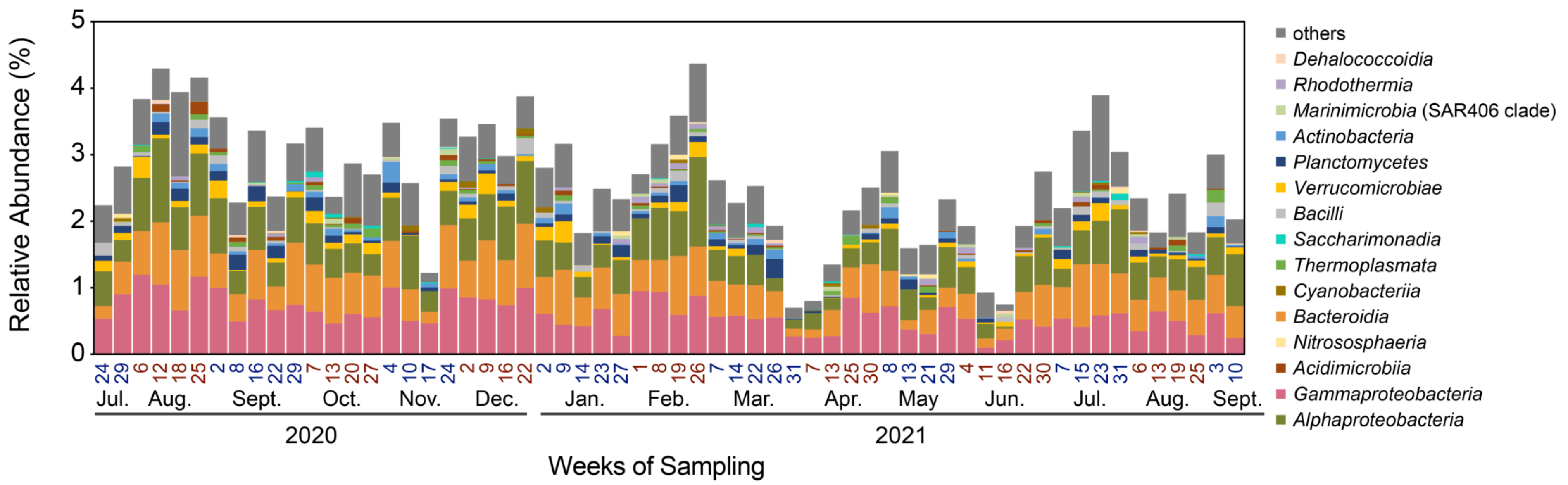

Fig. S4. Detailed community composition of the rare subcommunity.

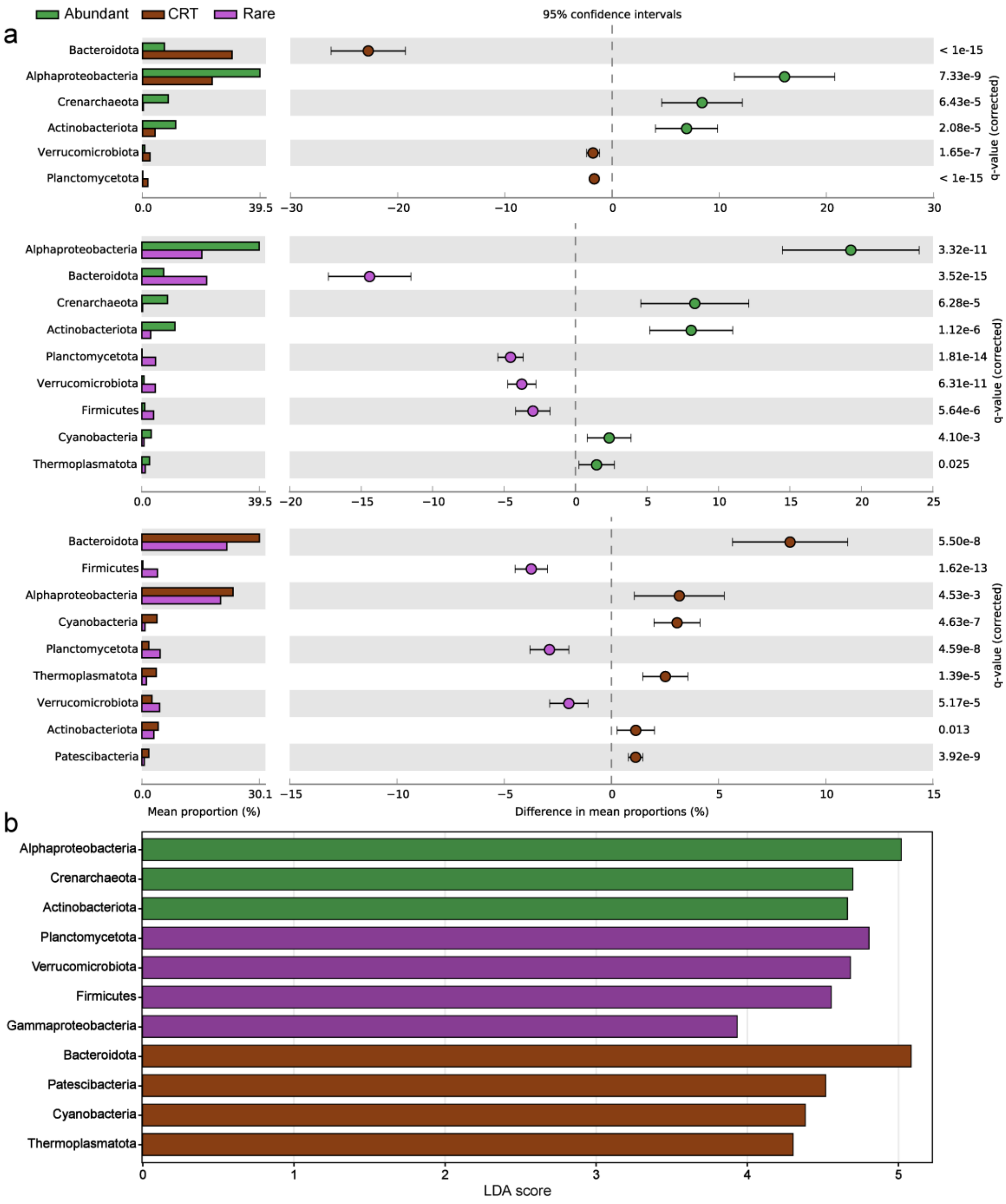

Fig. S5. Discriminant taxa in the three subcommunities. The taxa are ordered by effect sizes. (a) The STAMP analysis between each two subcommunities. The significance was calculated with Welch's test (Benjamini-Hochberg adjusted). (b) LefSe discoverring biomarkers with the linear discriminant analysis (LDA) with effect size. The default significance threshold (0.05) was set to select taxa for LefSe, as well as an LDA higher than 3.5.

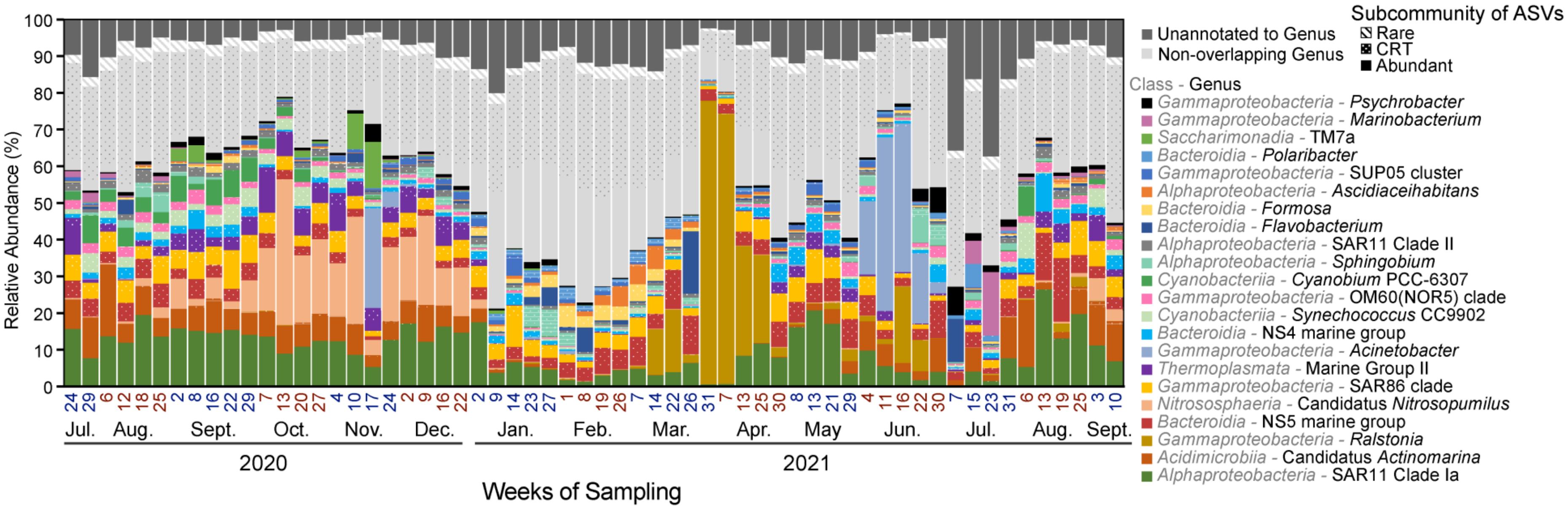

Fig. S6. The relative abundance of genera shared across all three subcommunities. Genera are distinguished by colors, while ASVs from different subcommunities are differentiated by textures. Solid bars: ASVs from abundant subcommunity; Dotted bars: ASVs from CRT subcommunity; Striped bars: ASVs from rare subcommunity; Dark grey: ASVs unannotated at the genus level; Light grey: genera not shared across all three subcommunities.

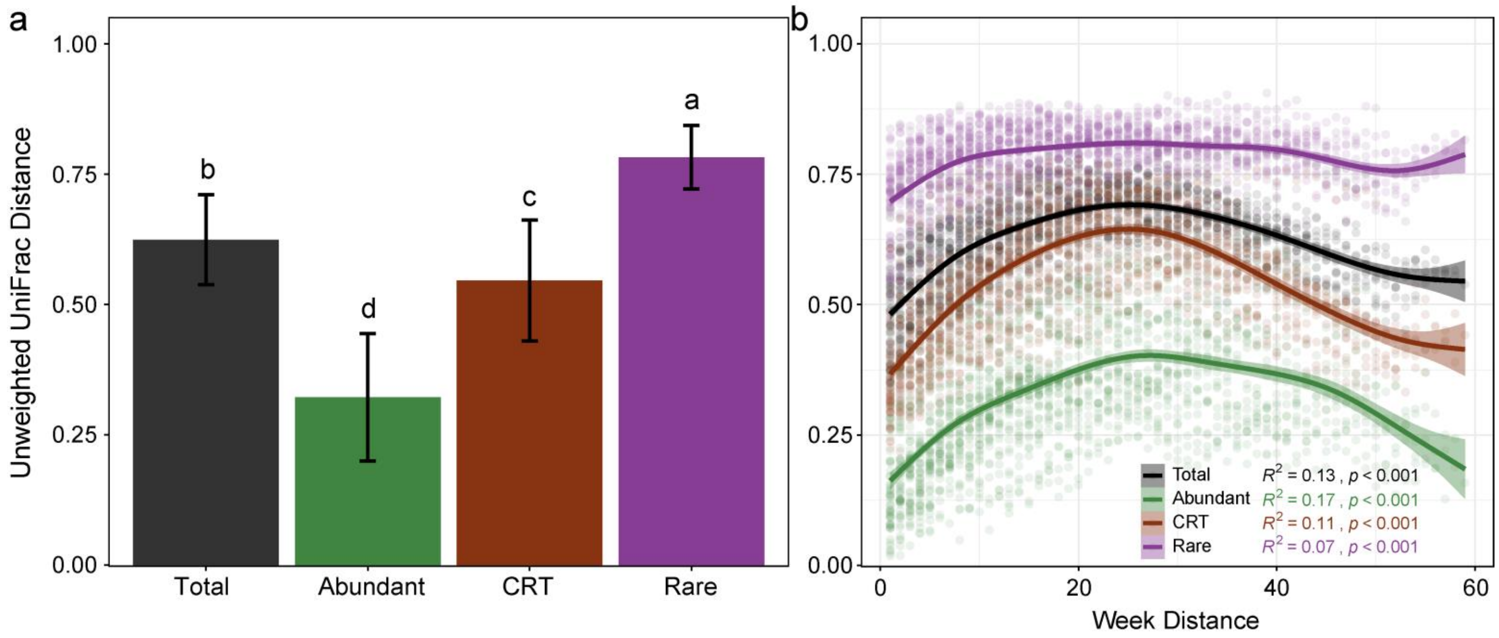

Fig. S7. Unweighted UniFrac distance of the different subcommunities. (a) Wilcoxon rank-sum tests were carried out to examine the significance of differences between groups; (b) Correlations between unweighted UniFrac and time distances at the ASV level in the three subcommunities and the total community. The correlation was fitted with a generalized additive model (GAM), with the shaded areas representing 95% confidence intervals. Spearman's correlation coefficients (r) and p values were used to evaluate the significance of the correlation between week distance and unweighted UniFrac distances.

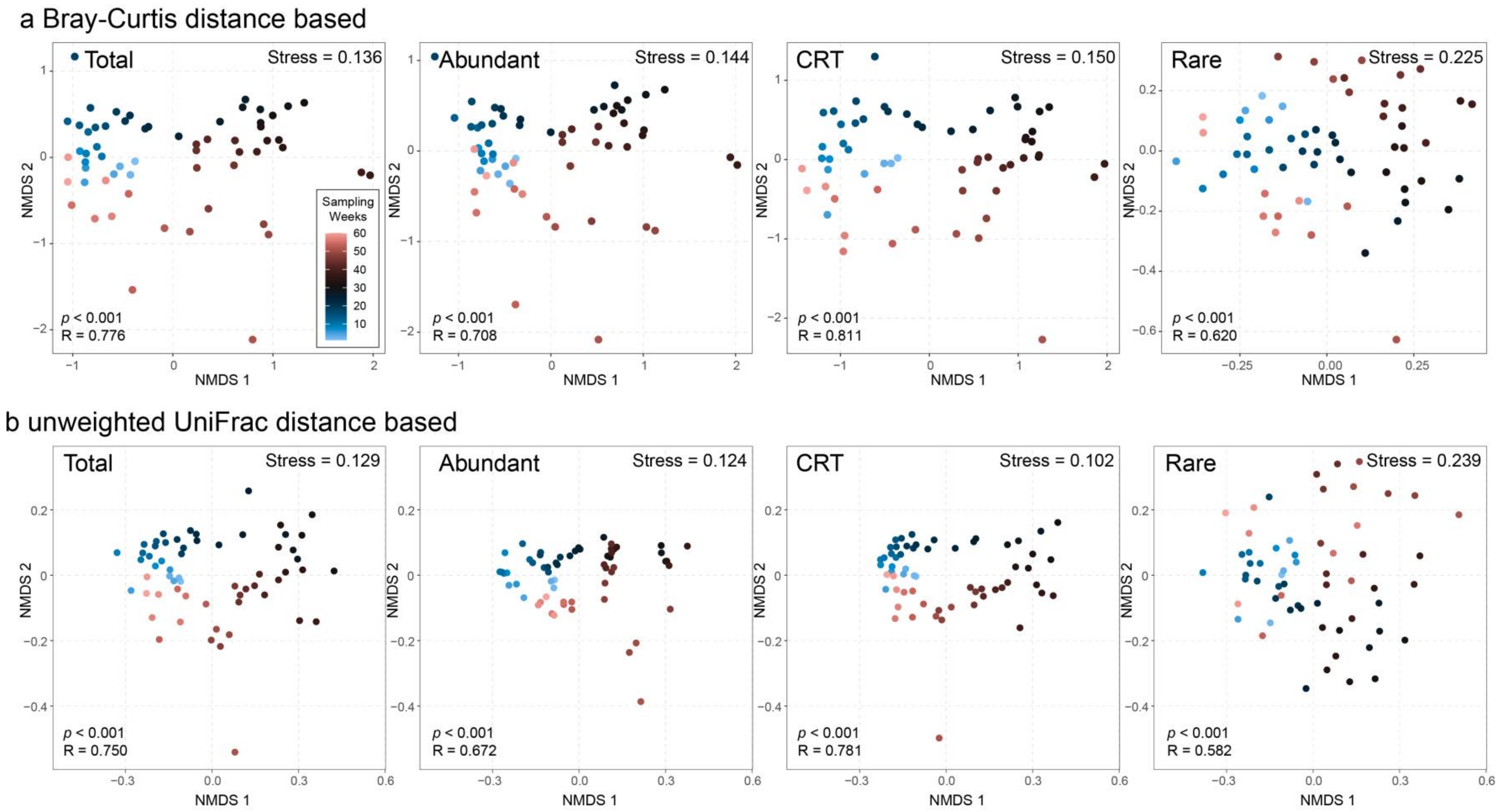

Fig. S8. Nonmetric multidimensional scaling ordination of abundant, CRT, rare subcommunities and total communities based on Bray-Curtis (a) and unweighted UniFrac (b) distances.

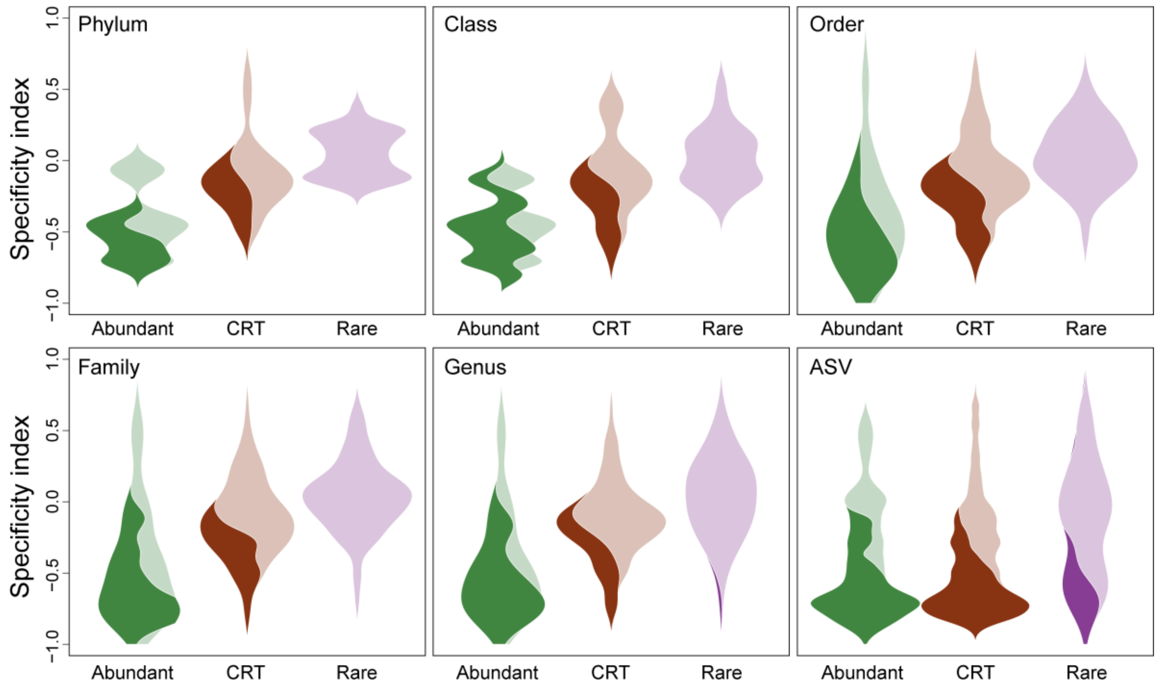

Fig. S9. The time specificity at multiple taxonomy levels in abundant, CRT, and rare subcommunities. The index varies between -1 and 1, with the lower value representing a stronger time specificity. Violin area is divided between species with statistically significant specificity (dark) versus species without specificity (light).

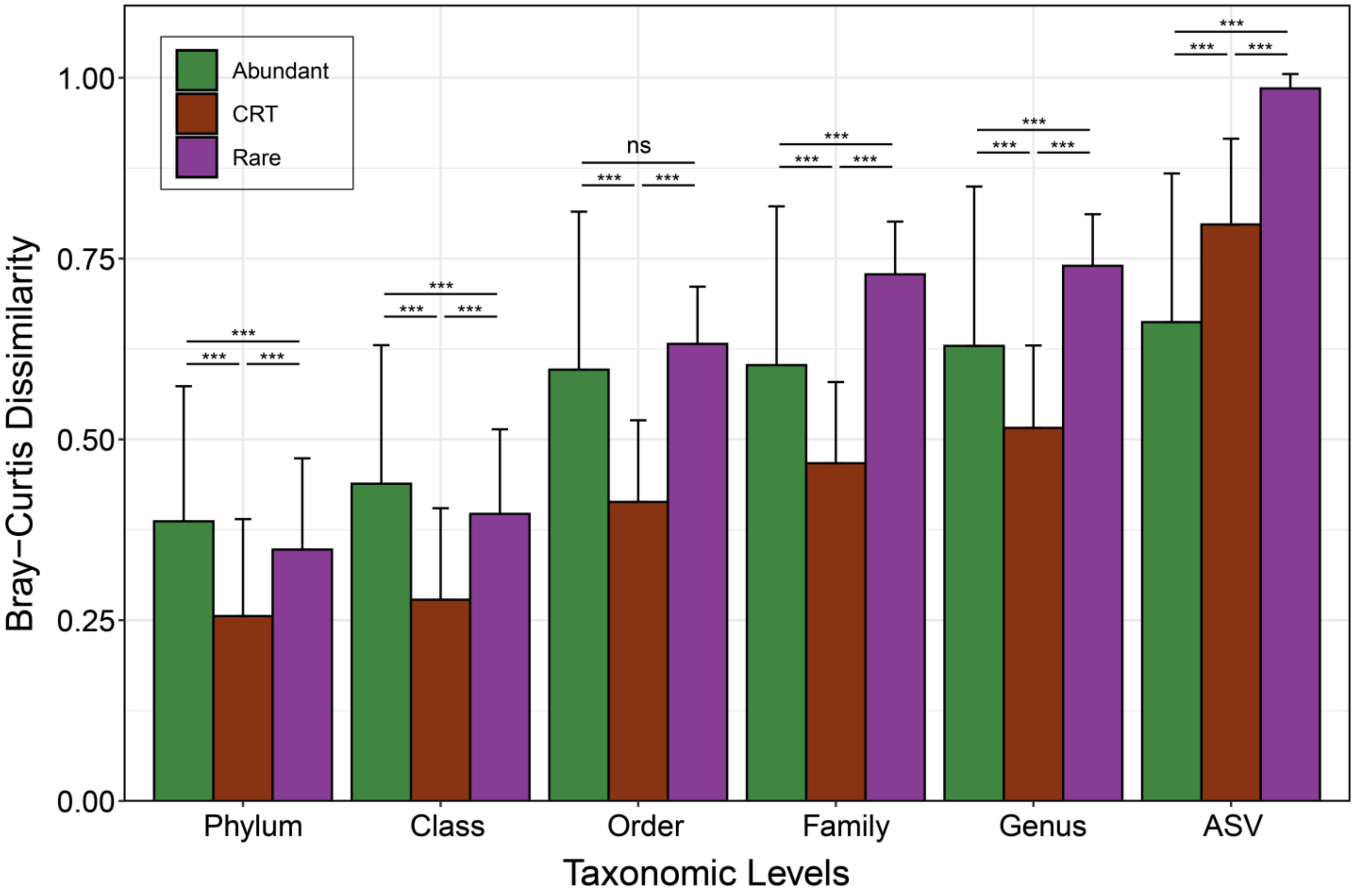

Fig. S10. The Bray-Curtis dissimilarity at multiple taxonomy levels in the three subcommunities. ***p <0.001, ns: not significant (Wilcoxon rank-sum tests).

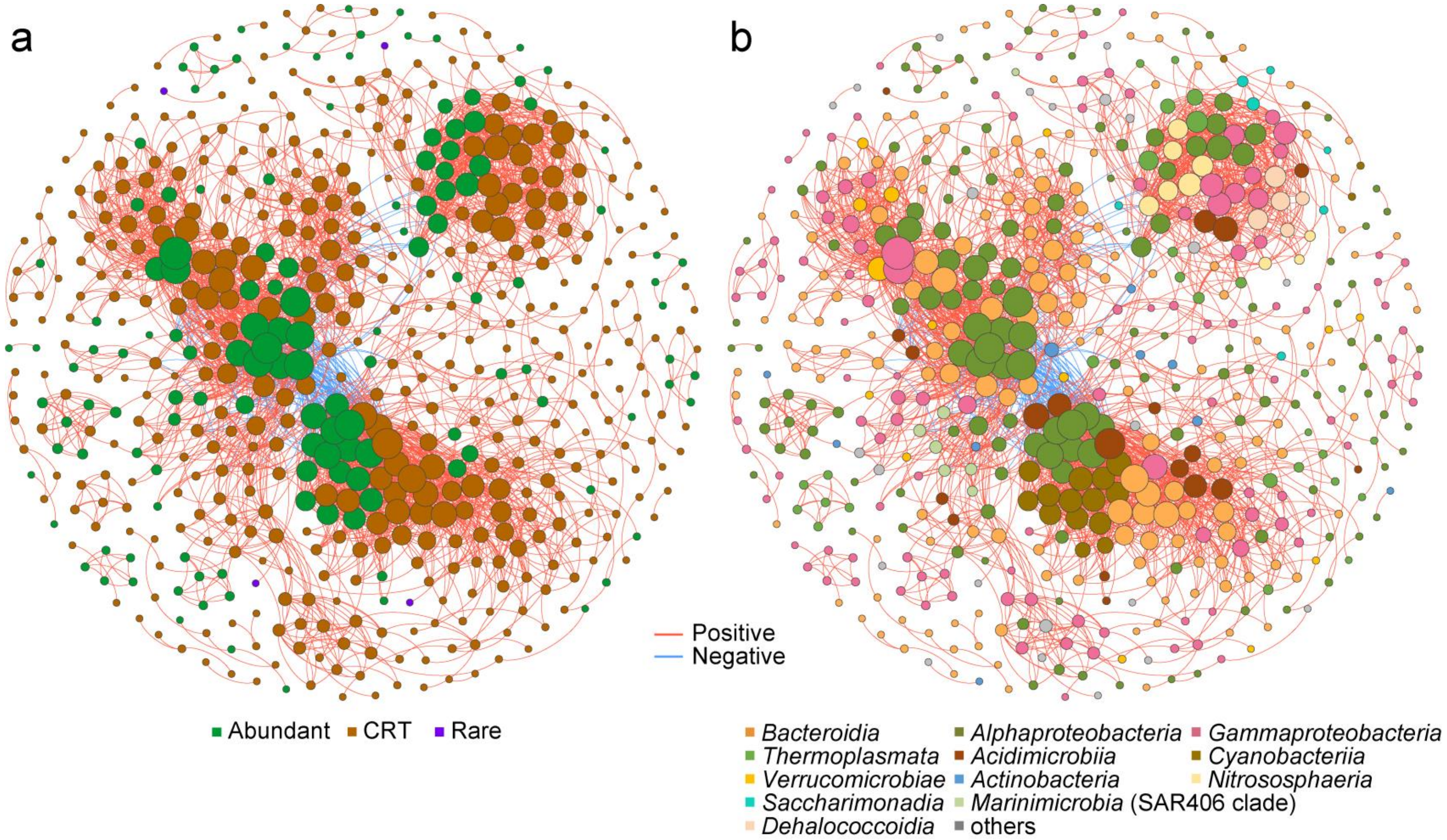

Fig. S11. Co-occurrence networks of the three subcommunities at ASV level. ASVs occurred in over 10 % of samples are involved in the networks. Edges are selected against the threshold of a correlation coefficient >|0.7| and a *p* value < 0.01. The size of each node is proportional to the number of correlations. (a) The nodes are colored by subcommunity. (b) The nodes are colored by class.

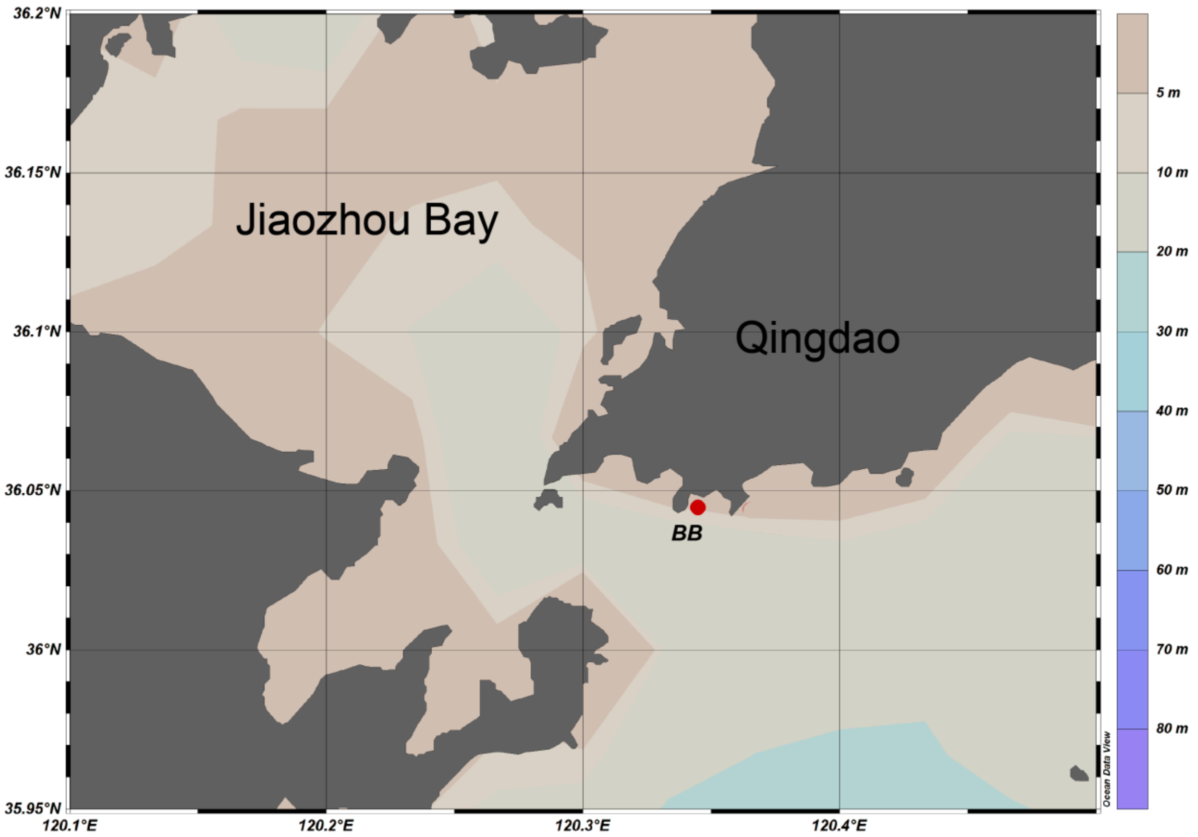

Fig. S12. Sampling map of the BB site in the coast of Qingdao. BB, Qingdao No. 2 Bathing Beach.

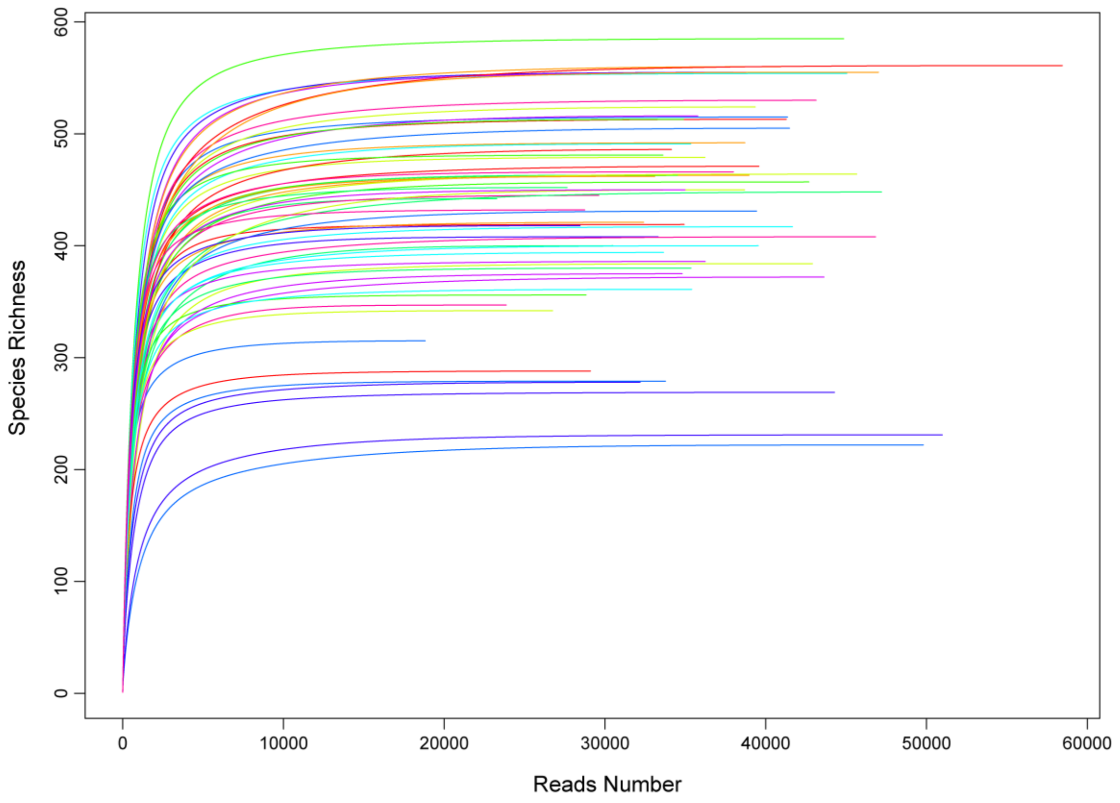

Fig. S13. The rarefaction curves of all samples.

Table S1. Statistical data of environmental factors.

(Table shown in separate file “Supplementary Table 1.xlsx”)

Table S2. Statistical data of abundant, CRT, and rare subcommunities.

	Abundant	CRT	Rare
Broad	14 (15.02 %)	15 (3.37 %)	0 (0.00 %)
Intermediate	149 (33.58 %)	806 (31.04 %)	21 (0.01 %)
Narrow	34 (2.29 %)	1838 (11.67 %)	3961 (2.95 %)
Sum	197 (50.89 %)	2659 (46.08 %)	3982 (3.05 %)

*ASV number (Relative abundance)

Table S3. Mantel test for total, abundant, CRT, and rare communities and environmental factors.

	Total	Abundant	CRT	Rare
pH	0.029	-0.009	0.055	0.056
Temperature	0.471***	0.438***	0.477***	0.353***
Chl a	0.087	0.084	0.092*	0.121***
NH_4^+	-0.010	-0.015	-0.021	0.026
NO_3^-	0.383***	0.366***	0.354***	0.256***
SiO_3^{2-}	0.512***	0.487***	0.501***	0.339***
NO_2^-	0.306***	0.242**	0.377***	0.217***
PO_4^{3-}	0.239**	0.224**	0.244***	0.138***
Salinity	0.022	-0.020	0.077	0.076*
DOC	0.072	0.078	0.072	-0.022
DIN	0.387***	0.373***	0.344***	0.253***

*: $p < 0.05$; **: $p < 0.01$; ***: $p < 0.001$

Table S4. Information for the 6,838 ASVs.

(Table shown in separate file “Supplementary Table 4.xlsx”)
